# Clinical impact of inappropriate DOAC dosing in atrial fibrillation: Insights from a real-world registry

**DOI:** 10.1016/j.ijcha.2025.101598

**Published:** 2025-01-03

**Authors:** Mustafa Yildirim, Hauke Hund, Matthias Mueller-Hennessen, Hugo A Katus, Norbert Frey, Evangelos Giannitsis, Christian Salbach

**Affiliations:** a Department of Internal Medicine III, Cardiology, University Hospital of Heidelberg, Germany; b DZHK (German Centre for Cardiovascular Research), Partner Site Heidelberg/Mannheim, Heidelberg, Germany

**Keywords:** Atrial fibrillation, Real-world evidence, Registry, Direct oral anticoagulants

## Abstract

**Background:**

A significant number of patients with atrial fibrillation (AF) on direct oral anticoagulants (DOACs) receives off-label or inappropriate doses. This study examines the prevalence, dosages, and clinical outcomes in AF-patients on DOAC therapy admitted to an emergency department (ED).

**Methods:**

This retrospective single-center observational study utilized data from the Heidelberg Registry of Atrial Fibrillation (HERA-FIB), consecutively including patients with AF presenting to the ED of the University Hospital of Heidelberg from June 2009 to March 2020. Rates of DOAC dosages at discharge from the ED were correlated with outcomes, focusing on a composite endpoint that included all-cause mortality, stroke, major bleeding, and myocardial infarction (MI).

**Resultsand Conclusions:**

Among 10,222 patients included in the HERA-FIB registry, 4,239 (41.5 %) were prescribed DOACs, and 3,031were eligible for the analysis. Of these, 2,199 (72.6 %) received appropriate dosages, 627 (20.7 %) were under-dosed, and 205 (6.8 %) were over-dosed. Under-dosed AF-patients demonstrated a significantly increased risk of the composite endpoint compared to those receiving appropriate dosages (HR 1.84, 95 %CI:1.55–2.18, p < 0.0001). Over-dosage had no significant effect on the HR for the composite endpoint, all-cause mortality, stroke, MI, or major bleeding compared to correct dosing but was associated with higher risks of the composite endpoint (HR 1.43, 95 %CI:1.04–1.96, p = 0.029) relative to under-dosage. This study underscores the critical importance of accurate DOAC dosing in patients with AF presenting to an ED. Both under-dosing and over-dosing are linked to significant clinical risks, highlighting the urgent need for improved dosing protocols and careful monitoring to enhance patient outcomes.

## 1 Introduction

Atrial fibrillation (AF) is a major contributor to embolic stroke, increasing the risk by five times [1]. AF-related strokes constitute about one-third of all ischemic strokes and tend to result in higher rates of mortality and long-term disability compared to other stroke etiologies [2], [3], [4]. Although oral anticoagulants (OACs) can reduce the stroke risk in AF patients by nearly 66 %, there is still significant underuse, with less than half of high-risk individuals receiving these therapies, including both vitamin K antagonists (VKAs) and direct oral anticoagulants (DOACs) [5], [6], [7], [8], [9]. Landmark trials such as RE-LY, ARISTOTLE, ROCKET-AF, and ENGAGE AF-TIMI 48 have established the safety and efficacy of DOACs, demonstrating their superiority or non-inferiority to VKAs in preventing stroke and systemic embolism while reducing bleeding risks [10], [11], [12], [13]. Inadequate anticoagulation is commonly observed in patients with AF who suffer an acute ischemic stroke [14]. The 2024 European Society of Cardiology (ESC) and 2023 American Heart Association/American College of Cardiology (AHA/ACC) guidelines emphasize the importance of initiating appropriate anticoagulation in AF patients based on thromboembolic and bleeding risk, with a focus on using DOACs over VKAs in most cases due to their improved safety and ease of use [15], [16]. The emergency department (ED) is highlighted as a critical setting for identifying patients with newly diagnosed AF, initiating anticoagulation, and managing AF in its early stages [17]. Previous research has mainly compared the safety profiles of individual DOACs with traditional warfarin [18]. Additionally, the applicability of clinical trial findings on DOACs to real-world patient populations has also been a topic of debate [19]. To date, there have been no head-to-head clinical trials comparing different types of DOACs, and limited data are available on the characteristics of patients on OACs presenting to the ED for any reason [20]. Additionally, there is a lack of comprehensive information regarding appropriate dosing and contraindication management. In response to these gaps, we conducted a retrospective analysis of AF patients admitted to our ED, evaluating the appropriateness of anticoagulant dosing based on manufacturer guidelines, patient characteristics, and outcomes including all-cause mortality, stroke, major bleeding, and myocardial infarction (MI), using data from a large, single-center registry.

## 2 Methods

### 2.1 Study population and design

**Fig. 1:**
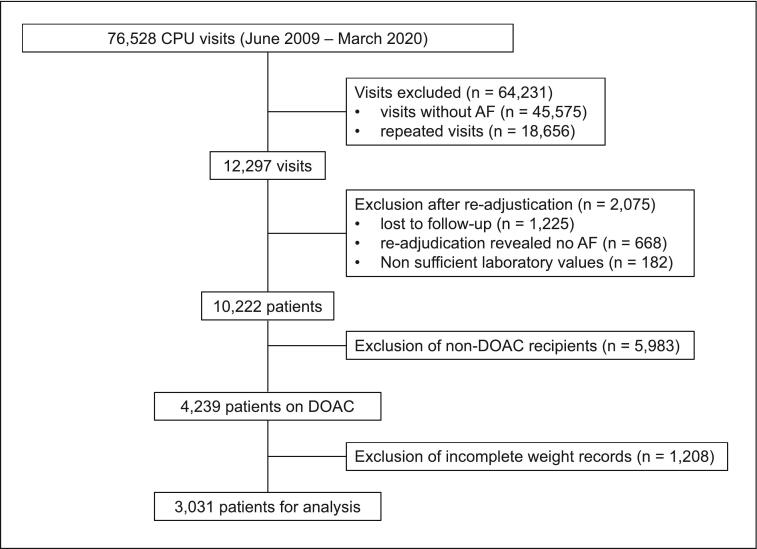
**Patient recruitment and selection flow diagram.** This flow diagram illustrating the recruitment and selection process for study participants, including the applied inclusion and exclusion criteria. **Abbreviations:** AF, atrial fibrillation; CPU, chest pain unit; DOAC, direct oral anticoagulants.

This analysis uses data from the Heidelberg Registry of Atrial fibrillation (HERA-FIB). The data collection and study population of this retrospective single-center study has already been described [21]. AF patients included within this registry were consecutively admitted to the ED of the University Hospital of Heidelberg from June 2009 to March 2020. Inclusion criteria comprised age ≥18  years and a confirmed diagnosis of AF at the time of presentation to the ED, either as primary reason for admission or as a comorbidity. Exclusion criteria were patients lost to follow-up for all-cause mortality, patients for whom re-adjudication revealed no AF, those with insufficient laboratory values for analysis, non-DOAC recipients, and patients with incomplete weight records. The patient selection process for this study is illustrated in Fig. 1**.** Medical decisions including treatment regimens or indication for oral anticoagulation of the patients were left to the decision of the treating physician. There was no impact on patient treatment owing to the retrospective observational design of the study. This study was approved by the local ethics committee of the Medical Faculty of Heidelberg. Informed consent for this retrospective analysis was waived by the local ethics committee. This study was conducted according to ethical principles stated in the Declaration of Helsinki. HERA-FIB is registered at ClinicalTrials.gov. ClinicalTrials.gov identifier: NCT05995561.

### 2.2 Follow-up and data

In order to ensure high follow-up rates, a sequential follow-up method was applied as described earlier [21]. Initially, data from the electronical hospital information system and hospital files from other hospitals affiliated with the University Hospital of Heidelberg were screened for information on outcome variables. Afterwards, structured patient phone calls were executed; if not possible, postal queries with standardized questionnaires were conveyed. If patients were still unattainable, registration offices were contacted, which could provide data on vital status and residency. If there was no information on outcome parameters such as stroke, major bleeding or MI, patients were excluded in analyses regarding these endpoints. The data that support the findings of this study are available from the corresponding author upon reasonable request.

### 2.3 Objectives

The objectives of this study were to evaluate the appropriateness of anticoagulant dosing in patients with AF presenting to the ED based on manufacturer guidelines, analyze patient characteristics associated with inappropriate dosing, and assess the clinical outcomes of under-dosing and over-dosing, including all-cause mortality, stroke, major bleeding, MI, and the composite endpoint comprising these events.

### 2.4 Definitions

A major bleeding event was defined according to the International Society on Thrombosis and Hemostasis (ISTH) major bleeding criteria [22]. Stroke definition excluded hemorrhagic stroke, which was allocated as a major bleeding event according to the ISTH major bleeding classification but included ischemic or unknown causes of stroke. Myocardial infarction was defined using the universal MI definition [23]. A composite endpoint for all-cause mortality, ischemic stroke, major bleeding and MI was assessed [21]. For the composite endpoint, patients were censored on the first event. Each patient's CHA_2_DS_2_-VASc score was assessed, with a score of 2 or higher indicating a need for OAC therapy as recommended by both the American Heart Association (AHA) and the European Society of Cardiology [17], [24]. Patients meeting this threshold are considered high-risk, as they face an annual thromboembolic event rate of 2.2 % [25]. Left ventricular ejection fraction (LV-EF) was assessed according to the guidelines recommended by the American Society of Echocardiography (ASE) and the European Association of Cardiovascular Imaging (EACVI) [26].

Participants were divided into two main groups based on DOAC dosing accuracy: one group with correct dosing and another with inappropriate dosing, which was further divided into underdosed and overdosed categories. Dosing was assessed according to the US Food and Drug Administration (FDA) guidelines: for Dabigatran, the standard dosage was 150 mg twice daily for patients with normal kidney function. A reduced dose of 110 mg twice daily was recommended for patients with a creatinine clearance (CrCl) of 15–30 mL/min, those over 80 years of age, or those taking concomitant P-glycoprotein inhibitors such as amiodarone, verapamil, quinidine, ketoconazole, clarithromycin, or ticagrelor. Dabigatran was not recommended for patients with a CrCl below 15 mL/min or for those with severe liver cirrhosis [27]. Rivaroxaban was indicated at 20 mg once daily for those without renal impairment, reduced to 15 mg daily for CrCl 15–50 mL/min, and avoided for CrCl below 15 mL/min or in cases of severe liver cirrhosis [28]. Apixaban was dosed at 5 mg twice daily, but reduced to 2.5 mg twice daily if the patient met at least two criteria: weight ≤ 60 kg, age ≥ 80 years, or serum creatinine ≥ 1.5 mg/dL or if CrCl 15–30 mL/min. Apixaban should be avoided for CrCl below 15 mL/min [29]. For Edoxaban, 60 mg once daily was prescribed for CrCl ≥ 51 mL/min, while 30 mg was used for CrCl 15–50 mL/min or if concomitant use of verapamil, dronedarone, ciclosporin, ketoconazol, erythromycin, or quinidine was given. The drug was contraindicated for CrCl under 15 mL/min [30]. The Cockcroft-Gault (C-G) equation was applied to calculate creatinine clearance [31]. Inappropriate dosing was defined as either underdosing (lower than recommended) or overdosing (higher than recommended). Supplementary Table 1 details the dosing guidelines and recommended adjustments for each medication.

### 2.5 Statistical analysis

Continuous variables were tested for normal distribution using the Kolmogorov-Smirnov test. Parametric data is presented as means (standard deviations, SD), non-parametric data as medians (25th, 75th percentiles, IQR). Comparisons between groups were conducted using chi-squared test or Fisher’s exact test for categorical variables and unpaired Student’s *t*-test or Wilcoxon rank-sum test for continuous variables. Kaplan-Meier analyses were performed to evaluate survival, and differences between groups were assessed using the log rank test. A multivariate logistic regression model was conducted to identify independent predictors of inappropriate dosing (overdose and underdose). The model included variables such as age, sex, renal function, and comorbidities. Interaction terms between key predictors were also evaluated to assess potential effect modifications. Additionally, a multivariate Cox proportional hazards model was employed to determine independent predictors for clinical outcomes, including all-cause mortality, stroke, major bleeding, and MI. The proportional hazards assumption for Cox models was tested using the Grambsch and Therneau method. Time-dependent receiver-operating-characteristic (ROC) curves from censored survival data using the Kaplan-Meier method were estimated and the area under the ROC curves (AUC) was calculated. The 95 % confidence interval (CI) of AUC was calculated according to Hanley and McNeil. A two tailed P-value of < 0.05 was considered to indicate statistical significance. Statistical analyses were performed using MedCalc Version 20.105 and R software (version 4.3.0, R Foundation for Statistical Computing, Vienna, Austria).

## 3 Results

### 3.1 Baseline characteristics of the entire cohort

**Fig. 2:**
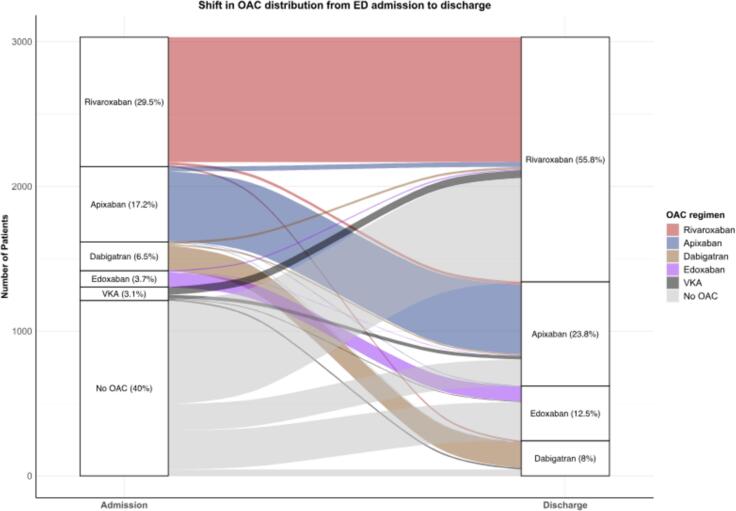
**Changes in oral anticoagulant (OAC) regimens during ED visits.** This figure depicts the distribution of oral anticoagulant (OAC) use among patients at admission to the ED and at discharge from the ED. The graph illustrates the changes in OAC use over the course of the ED visit. Abbreviations: ED, emergency department; OAC, oral anticoagulation; VKA, vitamin K antagonist.

A total of 10,222 patients were included within the HERA-FIB registry. Fig. 1 illustrates the patient selection process for the current analysis. After the exclusion of non-DOAC recipients and those with missing records, a total of 3,031 patients were included within the current analysis. Among these, 2,199 (72.6 %) received correct dosage, 205 (6.8 %) received over dosage and 627 (20.7 %) received under dosage. Fig. 2 shows the change of OAC distribution from admission to ED to discharge from ED. Baseline characteristics are reported stratified by DOAC dosage accuracy (Table 1). Briefly, the median age was 75 years (IQR 66.5–81), and 57.4 % of the cohort were male. The most prevalent comorbidity was arterial hypertension (83.2 %), followed by coronary artery disease (40.2 %) and diabetes mellitus (37.1 %). The median CHA_2_DS_2_-VASc score was 4 (IQR 3–5), indicating a moderate-to-high stroke risk, while the median HAS-BLED score was 2 (IQR 1–3), reflecting a mild to moderate bleeding risk. The distribution of DOAC use within included patients was varied, with Rivaroxaban being the most frequently prescribed (55.8 %), followed by Apixaban (23.8 %), Edoxaban (12.5 %), and Dabigatran (8.0 %).

**Table 1:** Baseline characteristics of patient cohort stratified by DOAC dosage accuracy.

	**Al patients** **(n = 3,031)**	**Correct dosage** **(n = 2,199)**	**Over dosage** **(n = 205)**	**Under dosage** **(n = 627)**	P value*
Age, y, median (IQR)	75 (66.5–81)	73 (65–80)	80 (75–85)	77 (71–82)	<0.0001
Sex, male, n (%all)	1,741 (57.4 %)	1,252 (56.9 %)	76 (37.1 %)	413 (65.9 %)	<0.0001
BMI, kg/m^2^, median (IQR)	26.6 (23.9–30.6)	26.9 (24.2–30.7)	23.1 (20.9–26.0)	27.1 (24.5–31.1)	<0.0001
HR, bpm, median (IQR)	93 (74–123)	98 (78–121)	98 (78–121)	84 (70–107)	<0.0001
BP sys, median (IQR) (n = 3,014)	146 (132–160)	146 (132–160)	144 (127–156)	146 (131–160)	0.063
BP dia, median (IQR) (n = 3,014)	87 (77–98)	87 (78–99)	84 (73–95.3)	85 (77–95)	0.542
**History**					
Arterial hypertension, n (% all)	2,521 (83.2 %)	1,792 (81.5 %)	166 (81 %)	563 (89.8 %)	0.001
Diabetes mellitus, n (% all) (n = 3,012)	1,125 (37.1 %)	715 (33 %)	78 (38 %)	332 (53 %)	0.024
Former CAD, n (% all)	1,219 (40.2 %)	790 (35.9 %)	80 (39 %)	349 (55.7 %)	<0.0001
Former CABG, n (% all)	261 (8.6 %)	156 (7 %)	15 (7.3 %)	90 (14.4 %)	0.008
Former MI, n (% all)	436 (14.4 %)	278 (12.6 %)	27 (13.2 %)	131 (20.9 %)	0.014
Former COPD, n (% all)	289 (9.5 %)	176 (8 %)	24 (12 %)	89 (14 %)	0.367
Former liver cirrhosis, n (%all)	13 (0.4 %)	3 (0.1 %)	7 (3.4 %)	3 (0.5 %)	0.001
Former stroke/TIA, n (% all)	673 (22.2 %)	479 (21.8 %)	49 (23.9 %)	145 (23.1 %)	0.0164
Former PAD, n (% all) (n = 3,028)	211 (7.0 %)	132 (6.0 %)	20 (9.8 %)	59 (9.4 %)	0.0033
**Diagnostic work up**					
Normal LVEF, n (% all)	1,095 (49.8 %)	836 (38.0 %)	71 (34.6 %)	188 (30.0 %)	<0.001
Mildly reduced LVEF, n (% all)	475 (21.6 %)	323 (14.7 %)	36 (17.6 %)	116 (18.5 %)	<0.001
Moderate reduced LVEF, n (% all)	302 (13.7 %)	203 (9.2 %)	23 (11.2 %)	76 (12.1 %)	<0.001
Severely reduced LVEF, n (% all)	328 (14.9 %)	206 (9.4 %)	26 (12.7 %)	96 (15.3 %)	<0.001
New-onset AF, n (%all)	920(30.4 %)	717 (32.6 %)	83 (40.5 %)	120 (19.1 %)	<0.001
**Risk scores**					
CHA_2_DS_2_VASc-score, median (IQR)	4 (3–5)	4 (2–5)	4 (4–5)	5 (4–5)	0.471
HASBLED-score, median (IQR)	2 (1–3)	2 (1–2)	2 (2–3)	2 (2–3)	<0.0001
**Laboratory**					
Hs-cTnT, ng/L, median (IQR)	18 (10–32)	16 (9–27)	24 (12–40)	25 (15–46)	<0.001
Serum creatinine, mg/dl, median (IQR)	0.95 (0.79–1.19)	0.94 (0.78–1.13)	1.09 (0.83–1.5)	1.01 (0.8–1.3)	0.007
CrCL, ml/min., median (IQR)	70.4 (50.8–95.7)	75.6 (54.7–101.4)	43.8 (35.6–48.8)	65.3 (52.1–81.9)	<0.001
**OAC regimen**					
Rivaroxaban, n (% all)	1,690 (55.8 %)	1,251 (56.9 %)	121 (59.0 %)	318 (50.7 %)	
Dabigatran, n (% all)	243 (8.0 %)	149 (6.8 %)	15 (7.3 %)	79 (12.6 %)	
Apixaban, n (% all)	720 (23.8 %)	493 (22.4 %)	17 (8.3 %)	210 (33.5 %)	
Edoxaban, n (% all)	378 (12.5 %)	306 (13.9 %)	52 (25.4 %)	20 (3.2 %)	
Clopidogrel, n (%all)	11 (0.36 %)	2 (0.09 %)	0 (0 %)	9 (1.44 %)	

**Abbreviations**: BMI, body mass index; AF, atrial fibrillation; HR, heart rate; bpm, beats per minutes; BP blood pressure; sys, systolic; dia, diastolic; CAD, coronary artery disease; CABG, coronary artery bypass graft surgery; COPD, chronic obstructive pulmonary disease; CrCl, creatinine clearance; DOAC, direct oral anticoagulant; hs-cTnT, high sensitive troponin T, ICB, intracerebral bleeding; LVEF, left ventricular ejection fraction; MI, myocardial infarction; NTproBNP, n-terminal-pro brain natriuretic peptide; OAC, oral anticoagulation; PAD, peripheral artery disease; TIA, transient ischaemic attack; VKA, vitamin K antagonist. LVEF classification: normal LVEF (≥52 %), mildly reduced LVEF (41–51 %), moderately reduced LVEF (30–40 %), and severely reduced LVEF (<30 %). *The p-values were computed to compare baseline characteristics among the groups: correct dosage, over dosage, and under dosage, with values < 0.05 indicating significant differences.

### 3.2 Comparison of under-dosage vs. Correct dosage

Patients receiving under-dosage were notably older than those on the correct dosage (median age 77 vs. 73 years, p < 0.005), had higher CHA2DS2-VASc scores (median 5 vs. 4, p < 0.005), and were more likely to have arterial hypertension (89.8 % vs. 81.5 %, p < 0.005) and coronary artery disease (55.7 % vs. 35.9 %, p < 0.005). Additionally, under-dosed patients had worse left ventricular function, with more presenting severely reduced LVEF (15.3 % vs. 9.4 %, p < 0.0001). The under-dosage group also had lower heart rates (median 84 vs. 98 bpm, p < 0.005), and higher hs-cTnT levels (25 vs. 16 ng/L, p < 0.005), reflecting a potentially higher burden of cardiovascular disease.

### 3.3 Comparison of over-dosage vs. Correct dosage

Patients in the over-dosage group were notably older than the correct-dosage group (80 vs. 74 years) and showed significantly lower eGFR values (median 43.8 vs. 74.6 ml/min, p < 0.001), reflecting poorer renal function. They also had a higher prevalence of diabetes (38 % vs. 33 %, p < 0.05), and slightly more reduced LVEF (severely reduced in 12.7 % vs. 9.4 %, p < 0.0001). However, there was a notable shift in DOAC prescription patterns among over-dosed patients compared to correctly dosed patients, with a higher proportion of over-dosed patients receiving Edoxaban (25.4 % vs. 13.9 %), a similar proportion receiving Rivaroxaban (59 % vs. 56.9 %), and a lower proportion receiving Apixaban (8.3 % vs. 22.4 %).

### 3.4 Comparison of over-dosage vs. under-dosage

Comparing over-dosed and under-dosed patients reveals that the under-dosed group was older (median age 77 vs. 80 years, p < 0.05), had worse cardiovascular profiles, including higher CHA2DS2-VASc scores (median 5 vs. 4, p < 0.005), and more severe comorbidities like arterial hypertension (89.8 % vs. 81 %, p < 0.005) and coronary artery disease (55.7 % vs. 39 %, p < 0.005). On the other hand, the over-dosage group had significantly worse renal function, with a median eGFR of 43.8 ml/min compared to 65.3 ml/min (p < 0.005). Additionally, the choice of DOACs differed substantially: the over-dosage group was more likely to receive Edoxaban (25.4 % vs. 3.2 %), while the under-dosage group received Apixaban more frequently (33.5 % vs. 8.3 %).

### 3.5 Predictors of inappropriate dosing

A multivariable logistic regression analysis identified several significant predictors of inappropriate DOAC dosing (Table 2). Increasing renal function (eGFR, OR 1.047, 95 % CI 1.029–1.065, p < 0.001) and higher weight (OR 1.063, 95 % CI 1.031–1.096, p < 0.001) were associated with an increased likelihood of inappropriate dosing. Conversely, first diagnosis of AF in the ED was associated with a lower likelihood of inappropriate dosing (OR 0.417, 95 % CI 0.271–0.772, p = 0.002). An interaction term between age and eGFR demonstrated a significant combined effect on inappropriate dosing (OR 1.003, 95 % CI 1.002–1.004, p < 0.001), suggesting that renal function's impact on dosing accuracy changes with advancing age.

**Table 2:** Multivariable logistic regression analysis predicting inappropriate dosing (overdosing or underdosing).

**Variable**	**Coefficient (β)**	**OR**	**95 % CI for OR**	**p-value**
Intercept	−8.219	−	−	<0.001
Age in years	0.027	1.028	0.992–1.065	0.135
eGFR (C-G)	0.046	1.047	1.029–1.065	<0.001
Sex (Male)	0.182	1.199	0.756–1.771	0.565
Weight in kg	0.061	1.063	1.031–1.096	<0.001
Hypertension	0.158	1.171	0.710–1.665	0.708
Diabetes Mellitus	−0.368	0.692	0.409–1.262	0.230
CAD	0.199	1.220	0.693–1.893	0.491
hs-cTnT	0.00020	1.000	0.997–1.004	0.863
NT-proBNP	−0.00001	1.000	0.999–1.000	0.359
New-Onset AF	−0.875	0.417	0.271–0.772	0.002
CHA2DS2-VASc Score	0.105	1.111	0.900–1.464	0.387
**Interaction Term**				
Age × eGFR (CG)	0.00255	1.003	1.002–1.004	<0.001

This table presents the results of a multivariable logistic regression analysis examining factors associated with inappropriate DOAC dosing. The odds ratios (ORs) with 95 % confidence intervals (CIs) represent the likelihood of overdosing or underdosing relative to correct dosing, adjusted for age, renal function, weight, and other clinical variables. An interaction term between age and eGFR (Cockcroft-Gault) was included to evaluate their combined effect. **Abbreviations:** AF, atrial fibrillation, CAD, coronary artery disease; C-G, Cockcroft-Gault; CI, confidence interval; eGFR, estimated glomerular filtration rate; hs-cTnT, high-sensitivity cardiac troponin T; kg, kilogram; NT-proBNP, N-terminal pro-brain natriuretic peptide.

### 3.6 Clinical outcomes associated with DOAC dosage accuracy

**Fig. 3:**
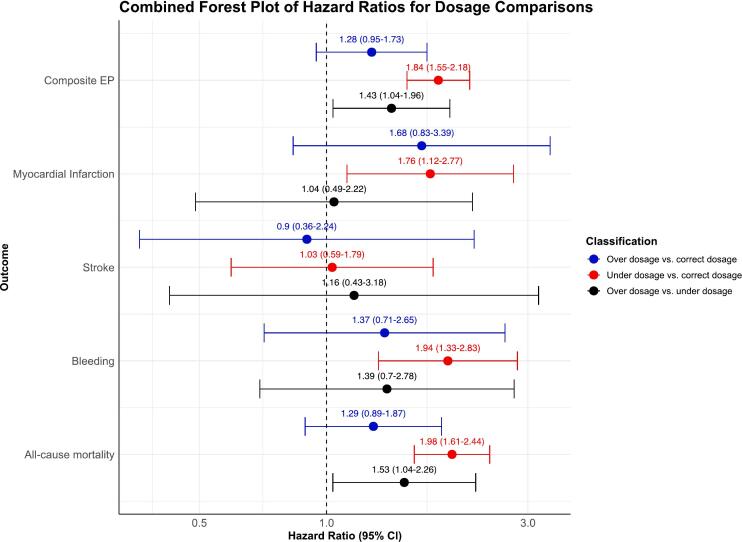
**Impact of DOAC dosage accuracy on clinical outcomes**. This figure presents a forest plot summarizing the hazard ratios (HRs) and their 95% confidence intervals (CIs) for clinical outcomes, including all-cause mortality, stroke, major bleeding events, myocardial infarction, and a composite endpoint, in relation to the accuracy of direct oral anticoagulant (DOAC) dosing. The plot visually compares the risk of these events across different levels of DOAC dosage accuracy.

**Fig. 4:**
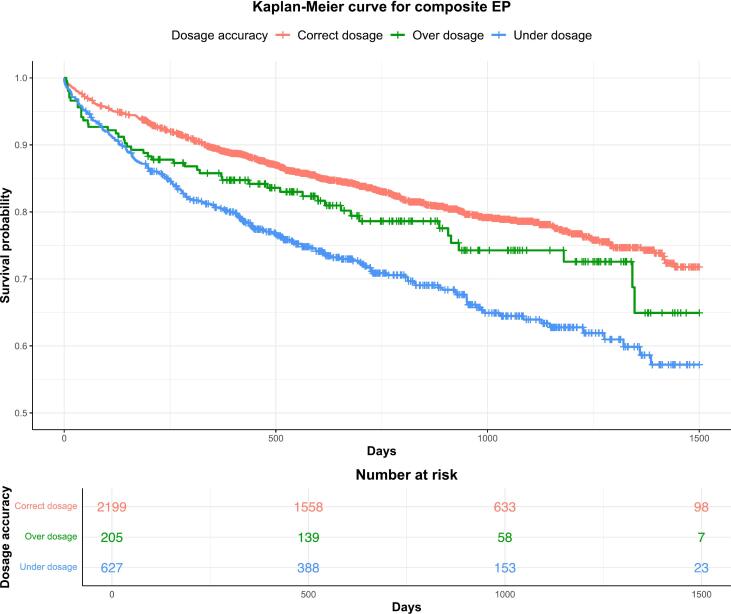
**Kaplan-Meier curves for composite endpoint by DOAC dosage.** This figure depicts Kaplan-Meier curves illustrating the survival differences over time among patients receiving direct oral anticoagulants (DOACs) with varying levels of dosage accuracy for the composite endpoint. The curves demonstrate that under-dosed patients have the poorest survival outcomes, followed by over-dosed patients, while correctly dosed patients exhibit the best survival.

A forest plot and hazard ratios (HRs) with 95 % CIs for outcomes (all-cause mortality, stroke, major bleeding, MI, and the composite endpoint) according to DOAC dosage accuracy are shown in Fig. 3. Under-dosed patients had a significantly higher risk of all-cause mortality compared to those on correct dosage (HR 1.98, 95 % CI 1.62–2.44, p < 0.0001). Over-dosed patients had a significantly higher risk of all-cause mortality compared to under-dosed patients (HR 1.53, 95 % CI 1.04–2.26, p = 0.033). Importantly, under-dosage was also associated with increased risks of major bleeding compared to correctly dosed patients (HR 1.94, 95 % CI 1.33–2.83, p = 0.0006). Additionally, under-dosage was associated with increased risks of MI (HR 1.76, 95 % CI 1.12–2.77, p = 0.0146). These elevated risks may reflect the overall higher cardiovascular burden observed in the under-dosed group. In contrast, over-dosage did not significantly increase the risk of all-cause mortality (HR 1.29, 95 % CI 0.89–1.87, p = 0.1781), stroke (HR 0.90, 95 % CI 0.36–2.24, p = 0.8181), or the composite endpoint (HR 1.28, 95 % CI 0.95–1.73, p = 0.1095) compared to correct dosage. While the bleeding risk with over-dosage was higher compared to correct dosage, this difference was not statistically significant (HR 1.37, 95 % CI 0.71–1.73, p = 0.3446). However, over-dosage was associated with a significantly higher risk of all-cause mortality relative to under-dosage (HR 1.53, 95 % CI 1.04–2.26, p = 0.0328) and an increased risk of the composite endpoint (HR 1.43, 95 % CI 1.04–1.96, p = 0.0293), For the composite endpoint, under-dosage significantly increased the risk compared to correct dosage (HR 1.84, 95 % CI 1.55–2.18, p < 0.0001).Table 3 provides a detailed analysis of the HRs for each outcome, including 95 % CIs. Kaplan-Meier curves for the composite endpoint (Fig. 4) illustrate the survival differences over time between the dosage groups, with the under-dosed group showing the poorest outcomes, followed by the over-dosed and correctly dosed groups. These curves visually complement the hazard ratios, offering insight into how survival probabilities diverge over time.

**Table 3:** Hazard ratios of OAC dosage accuracy on clinical outcomes in patients with atrial fibrillation.

**Outcome**	**Classification**	**Hazard Ratio** **(95 % CI)**	**P-value**
**All-cause mortality**			
	Over dosage vs. correct dosage	1.29 (0.89–1.87)	0.1781
	Under dosage vs. correct dosage	1.98 (1.62–2.44)	<0.0001
	Over dosage vs. under dosage	1.53 (1.04–2.26)	0.0328
**Major bleeding event**			
	Over dosage vs. correct dosage	1.37 (0.71–2.65)	0.3446
	Under dosage vs. correct dosage	1.94 (1.33–2.83)	0.0006
	Over dosage vs. under dosage	1.39 (0.70–2.78)	0.3511
**Stroke**			
	Over dosage vs. correct dosage	0.90 (0.36–2.24)	0.8181
	Under dosage vs. correct dosage	1.03 (0.59–1.79)	0.9122
	Over dosage vs. under dosage	1.16 (0.43–3.18)	0.7694
**Myocardial Infarction**			
	Over dosage vs. correct dosage	1.68 (0.83–3.39)	0.1464
	Under dosage vs. correct dosage	1.76 (1.12–2.77)	0.01460
	Over dosage vs. under dosage	1.04 (0.49–2.22)	0.9151
**Composite EP**			
	Over dosage vs. correct dosage	1.28 (0.95–1.73)	0.1095
	Under dosage vs. correct dosage	1.84 (1.55–2.18)	<0.0001
	Over dosage vs. under dosage	1.43 (1.04–1.96)	0.0293

**Abbreviations**: EP, endpoint.

## 4 Discussion

AF significantly elevates the risk of stroke, increasing it by approximately five times, while oral anticoagulation can reduce this risk by over two-thirds and lower all-cause mortality [5], [32], [33]. In clinical practice, a primary challenge is ensuring the correct dosing of anticoagulants, including both warfarin and direct oral anticoagulants (DOACs). Although the clinical impacts of DOAC under-dosing are less well-established, some studies suggest it may lead to an increased risk of stroke and mortality [34], [35]. This study seeks to further evaluate the clinical consequences of both appropriate and inappropriate DOAC dosing. In this retrospective analysis of a cohort of consecutive ED patients, we report several interesting findings. First, under-dosage of DOACs was associated with significantly worse clinical outcomes, including higher rates for all-cause mortality, major bleeding events, and MI, compared to correct dosage. These risks were particularly elevated for the composite endpoint, where under-dosed patients showed markedly poorer outcomes over time. Second, predictors of inappropriate DOAC dosing were identified through multivariable logistic regression analysis. Increased renal function (eGFR, OR 1.047, 95 % CI 1.029–1.065, p < 0.001) and higher weight (OR 1.063, 95 % CI 1.031–1.096, p < 0.001) were significantly associated with a higher likelihood of inappropriate dosing. Conversely, first diagnosis of AF in the ED reduced the likelihood of inappropriate dosing (OR 0.417, 95 % CI 0.271–0.772, p = 0.002). Furthermore, the interaction between age and eGFR (OR 1.003, 95 % CI 1.002–1.004, p < 0.001) underscored the complex relationship between renal function, age, and dosing accuracy, highlighting the nuanced challenges of tailoring anticoagulation therapy. Third, over-dosage was not significantly associated with an increased risk of all-cause mortality or stroke compared to correct dosing. While over-dosage demonstrated a non-significant increase in major bleeding risk relative to correct dosing and under-dosing, it was linked to significantly worse outcomes compared to under-dosage for all-cause mortality (HR 1.53, 95 % CI 1.04–2.26, p = 0.033) and the composite endpoint (HR 1.43, 95 % CI 1.04–1.96, p = 0.029). Finally, Kaplan-Meier survival curves reinforced these findings by visually demonstrating the cumulative disadvantage in survival for under-dosed patients across the follow-up period. Together, these results highlight the critical importance of achieving accurate DOAC dosing in atrial fibrillation patients.

In this study 27.4 % of patients on DOAC were inappropriately dosed according to FDA criteria, with the majority of these patients being underdosed (75.4 %) and 24.6 % being overdosed. The rate of inappropriate dosing observed in our study is notably high, falling at the upper range compared to findings in other studies (11 %–31 %) [35], [36], [37], [38], [39]. This elevated prevalence of under-dosing may be attributed to selection bias, as our tertiary referral center serves patients with complex medical conditions and a significant burden of comorbidities. Additionally, studies that rely on administrative data often lack detailed patient weight information, which can lead to misclassification of those deemed under-dosed, potentially skewing the study results [35], [37]. Our findings revealed a notably high likelihood of Edoxaban being overdosed in this cohort. This may be attributed to the drug’s simplified dosing scheme, which depends heavily on body weight and creatinine clearance thresholds, potentially leading to errors in dose adjustment [30]. To further explore predictors of inappropriate DOAC dosing, we conducted a multivariable logistic regression analysis. This revealed that renal function (eGFR), weight, and new-onset atrial fibrillation significantly influenced the likelihood of inappropriate dosing. Specifically, higher eGFR and weight were associated with increased odds of overdosing, while new-onset atrial fibrillation significantly decreased the odds of both overdosing and underdosing. Moreover, an interaction term between age and eGFR demonstrated a nuanced relationship, indicating that the effect of renal function on dosing decisions varies with age. These findings highlight the complexity of dosing decisions in clinical practice and underscore the need for tailored interventions, particularly for high-risk subgroups. Additionally, Edoxaban is less commonly prescribed than other DOACs such as Rivaroxaban, and provider unfamiliarity may contribute to dosing inaccuracies. While overdosing showed a trend towards increased bleeding risk compared to underdosing in our study (HR 1.39, 95 % CI 0.70–2.78, p = 0.35), this finding was not statistically significant. This highlights the need for targeted education and improved decision support tools to minimize dosing errors, particularly for less frequently used DOACs. A recent large-scale cohort study in Switzerland found that nearly 17 % of patients with atrial fibrillation on DOACs were prescribed off-label reduced doses [40]. Regression analysis indicated that concurrent aspirin use significantly increased the likelihood of bleeding (odds ratio 1.7 [1.0–2.8], P = 0.042) and was also linked to a higher risk of severe bleeding complications. In contrast, clopidogrel and the type of anticoagulation (VKA vs. DOAC) had no significant impact on bleeding risk or prevalence upon admission, nor did they affect in-hospital mortality. (40) Similarly, a study from Japan reported comparable rates of off-label DOAC dosing for stroke prophylaxis in AF but did not find an associated increase in stroke or mortality rates among patients receiving reduced doses [41]. Another study from Israel revealed that nearly 39 % of patients with atrial fibrillation on DOACs were prescribed off-label reduced doses, primarily due to concerns about bleeding risks. This study demonstrated that patients on these off-label reduced dosages experienced a significantly higher risk for a composite outcome of all-cause mortality, stroke, and MI, while their bleeding rates did not decrease [42]. The findings of the current study align with these results, indicating a substantial proportion of patients using off-label reduced doses of DOACs and a paradoxically higher bleeding risk for under-dosed patients compared to those receiving appropriate doses (HR = 1.94, CI: 1.33–2.83, p = 0.006). This paradoxical finding may reflect the underlying cardiovascular burden and comorbidities in these patients, which could contribute to adverse outcomes independent of anticoagulation. These findings challenge the notion that under-dosing is a viable strategy for mitigating bleeding risk. Instead, they underscore the importance of adhering to guideline-recommended dosing protocols to achieve optimal safety and efficacy [15], [16]. Furthermore, the under-dosed group shows consistently poorer outcomes throughout the follow-up period, followed by the over-dosed group, and the correctly dosed group. This visual representation emphasizes the persistent risk faced by under-dosed patients, aligning with the hazard ratio findings.

Our findings, along with those from the three studies mentioned, highlight a common observation in daily clinical practice: fear of bleeding complications leads physicians to prescribe DOACs at off-label reduced doses, which could potentially harm patients [40]. There is conflicting evidence regarding the effects of these off-label dose reductions on the rates of stroke and bleeding. In fact, data from real-world studies often show different results than those from initial studies funded by manufacturers. For example, in our study, the median CHA2DS2-VASc score was 4, increasing to 5 in the under-dosing group, while a mean score of 2 was reported in the atrial fibrillation trial comparing apixaban to warfarin [43]. In another trial comparing rivaroxaban with warfarin for nonvalvular atrial fibrillation, the mean CHA_2_DS_2_-VASc score was 3.5, which is much closer to our findings [44]. This suggests that manufacturers’ claims about lower bleeding rates for one DOAC compared to another are not very relevant unless direct comparison trials are conducted.

Furthermore, in our study, 6.8 % of patients were classified as over-dosed, a figure consistent with findings from other studies [38], [45]. Patients receiving over-doses of DOACs did not demonstrate a significant increase in the risk of all-cause mortality, stroke, major bleeding or the composite endpoint compared to those receiving appropriate dosages. This absence of difference may be attributed to similar characteristics between the two groups; both had comparable median ages (80 vs. 73 years), median CHA2DS2-Vasc scores of 4, and similar proportions of severe left ventricular ejection fraction abnormalities (12.7 % vs. 9.4 %, p < 0.0001). Interestingly, over-dosage was not associated with statistically significant major bleeding events compared to either under-dosage (HR 1.39, 95 % CI 0.70–2.78, p = 0.35) or correct dosage (HR 1.37, 95 % CI 0.71–2.65, p = 0.34). This finding is unexpected, as over-dosage is typically assumed to increase bleeding risk. One possible explanation is the relatively smaller sample size of the over-dosed group, which may have limited statistical power to detect significant differences. Additionally, the over-dosage group had the highest rates of Edoxaban use compared to the other two groups (25.4 % vs. 13.9 % in the correct-dosage group and 3.2 % in the under-dosage group), while having the lowest percentage of Apixaban users (8.3 % vs. 22.4 % in the correct-dosage group and 33.5 % in the under-dosage group). The simplified dosing scheme of Edoxaban may offer a degree of safety even at higher doses, potentially mitigating the bleeding risk associated with over-dosage. These factors, along with the smaller sample size, may explain the lack of statistically significant bleeding risk in the over-dosed group, although the precise reasons remain uncertain. The unequal distribution of these DOACs across dosage groups may have influenced the observed outcomes. At this time, we cannot provide further analysis on the implications of these prescription patterns; thus, a head-to-head comparison of DOACs regarding outcomes should be pursued in future prospective studies.

## 5 Limitations

This study is a retrospective single-center observational analysis of patients with AF presenting to the ED, and thus, our findings should be interpreted within the context of an acute clinical environment. Data for this registry were gathered from various sources, including electronic records, discharge summaries, structured phone interviews, questionnaires, and data from registration offices. Consequently, the possibility of human error may lead to incomplete or inaccurate data. The follow-up rate for patients was notably high, with over 90 % of participants' survival status ascertained, which is commendable for an observational retrospective study involving emergency patients and minimal exclusion criteria conducted over 11 years.

Furthermore, we opted not to conduct a sensitivity analysis comparing treatment benefits between patients newly initiated on direct oral anticoagulants (DOACs) due to the small sample size and low event rates in these groups. Patients receiving dual antiplatelet therapy, such as clopidogrel with oral anticoagulants, were not excluded; however, their representation in the cohort was very small (0.36 %), and a substantial number had to be excluded due to missing variables. This subgroup's limited size restricts generalizability and was reflected in the baseline characteristics.

We also did not account for drug discontinuation, which may introduce bias related to the cessation of oral anticoagulant therapy. Despite sequential follow-up within the HERA-FIB study, the retrospective nature of our analysis means that an underestimation of event rates cannot be entirely ruled out. Bleeding events were classified according to the International Society on Thrombosis and Haemostasis (ISTH) major bleeding criteria [22]; however, we lacked data on the underlying causes of these bleeding events, limiting our ability to differentiate between reasons for bleeding. Lastly, as is common in observational studies, particularly those conducted in ED settings, the potential for unmeasured or residual confounding that could impact our results remains.

## 6 Conclusions

In conclusion, our study provides valuable real-world data on the implications of dosing accuracy for DOACs in patients with AF. Under-dosage was associated with significantly worse clinical outcomes, including increased all-cause mortality, major bleeding events, and myocardial infarction. In contrast, over-dosage did not significantly affect all-cause mortality, stroke and major bleeding risk compared to correct dosing, although it was associated with a higher risk of the composite endpoint relative to under-dosage and a higher risk of all-cause mortality compared to under-dosage. Given that a substantial proportion of patients were inappropriately dosed, our findings underscore the necessity for improved dosing protocols and vigilant patient monitoring to optimize anticoagulant therapy and enhance patient outcomes. Future studies should further explore the effects of different DOACs on clinical outcomes, particularly in complex patient populations encountered in emergency settings.

## 7 Disclosures

**M.M−H.** received research funding from BRAHMS Thermo Fisher Scientific and Roche Diagnostics and served as a consultant to ZOLL CMS GmbH. **E.G.** received honoraria for lectures from Roche Diagnostics, AstraZeneca, Bayer, Daiichi-Sankyo, Lilly Eli Deutschland. He serves as a consultant for Roche Diagnostics, BRAHMS Thermo Fisher Scientific, Boehringer Ingelheim and has received research funding from BRAHMS Thermo Fisher Scientific, Roche Diagnostics, Bayer Vital and Daiichi Sankyo. **N.F.** has received speaker honoraria from Daiichi Sankyo, Astra Zeneca, Boehringer Ingelheim and Bayer Vital. There are no disclosures for **M.Y.**, **H.H.**, **H.A.K.** and **C.S**.

## CRediT authorship contribution statement

**Mustafa Yildirim:** Writing – review & editing, Writing – original draft, Visualization, Validation, Supervision, Software, Resources, Project administration, Methodology, Investigation, Formal analysis, Data curation, Conceptualization. **Hauke Hund:** Visualization. **Matthias Müller-Hennessen:** Visualization. **Hugo A Katus:** . **Norbert Frey:** Visualization, Validation. **Evangelos Giannitsis:** Writing – review & editing, Visualization, Validation, Supervision, Software, Resources, Project administration, Methodology, Investigation, Funding acquisition, Data curation, Conceptualization. **Christian Salbach:** Writing – review & editing, Visualization, Validation, Project administration, Methodology, Investigation, Funding acquisition, Data curation, Conceptualization.

## Declaration of competing interest

The authors declare the following financial interests/personal relationships which may be considered as potential competing interests: [The study was supported by a research grant from Daiichi Sankyo and Bayer diagnostics. The sponsor had no influence on the study concept, data collection, or interpretation. the funders had no role in study design, data collection and analysis, decision to publish, or preparation of the manuscript].
