# Supplementary material for: Clinical impact of inappropriate DOAC dosing in atrial fibrillation: Insights from a real-world registry
Source: Int J Cardiol Heart Vasc. 2025 Jan 3;56:101598. doi: 10.1016/j.ijcha.2025.101598 (PMC11758832; doi:10.1016/j.ijcha.2025.101598)
Supplement: Supplementary Data 1 [file mmc1.docx]

**Supplemental material**

**Supplement Table 1. DOAC dosage recommendations and contraindications for stroke prevention in AF patients.**

| **DOAC** | **Manufacturer´s recommendation** | **Recommended dosage reductions** | **Contraindication** |
| --- | --- | --- | --- |
| Rivaroxaban (1) | 20 mg once daily | 15 mg once daily if CrCl 15-50 mL/min | CrCl below 15 mL/min or in cases of severe liver cirrhosis |
| Apixaban (2) | 5 mg twice daily | 2.5 mg twice daily if 2 of following criteria present:   - age ≥80 years - weight ≤60 kg - serum creatinine ≥1.5 mg/dL or if CrCl 15-30 mL/min | CrCl below 15 mL/min |
| Edoxaban (3) | 60 mg once daily | 30 mg once daily if 1 or more of following criteria present:   - CrCl 15-50 mL/min - weight ≤60 kg - concurrent use of cyclosporin, dronedarone, erythromycin, ketoconazole, quinidine, or verapamil | CrCl below 15 mL/min |
| Dabigatran (4) | 150 mg twice daily | 110 mg twice daily if 1 or more of following criteria present   - CrCl 30-50 mL/min - age >80 years - concomitant P-glycoprotein inhibitors such as amiodarone, verapamil, quinidine, ketoconazole, clarithromycin, or ticagrelor | CrCl below 30 mL/min or in cases of severe liver cirrhosis |

**Abbreviations:** AF, atrial fibrillation; CrCl, creatinine clearance; DOAC, direct oral anticoagulant.

**References**

1. Janssen Pharmaceutical Companies. Rivaroxaban (Xarelto) [package insert]. U.S. Food and Drug Administration website [Available from: <https://www.accessdata.fda.gov/drugsatfda_docs/label/2023/022406s041lbl.pdf>.

2. Bristol-Myers Squibb Company. Apixaban (Eliquis) [package insert]. U.S. Food and Drug Administration website; [Available from: <https://www.accessdata.fda.gov/drugsatfda_docs/label/2021/202155s034lbl.pdf>.

3. Daiichi Sankyo Co. Edoxaban (Savaysa) [package insert]. U.S. Food and Drug Administration website [Available from: <https://www.accessdata.fda.gov/drugsatfda_docs/label/2015/206316lbl.pdf>.

4. Boehringer Ingelheim Pharmaceuticals. Dabigatran (Pradaxa) [package insert]. U.S. Food and Drug Administration website; [Available from: <https://www.accessdata.fda.gov/drugsatfda_docs/label/2024/022512s047lbl.pdf>.
